# Supplementary material for: Effect of prosocial public health messages for population behaviour change in relation to respiratory infections: a systematic review protocol
Source: BMJ Open. 2021 Jan 13;11(1):e044763. doi: 10.1136/bmjopen-2020-044763 (PMC7812082; doi:10.1136/bmjopen-2020-044763)
Supplement: Supplementary data [file bmjopen-2020-044763supp001.pdf]

Supplementary Table 1. Search strategy terms (Medline)

1. exp respiratory tract infections/

2. exp Coronavirus Infections/ or exp SARS Virus/ or exp Severe Acute Respiratory Syndrome/

3. exp Coronavirus/

4. exp Influenza A Virus, H1N1 Subtype/ or exp Influenza, Human/

5. (covid-19 or coronavir* or corona virus* or covid19 or covid 19 or coronavirus infection* or coronavirus or SARS virus or CoV2 or sarscov2 or Severe Acute Respiratory Syndrome or influenza or flu or H1N1 or Ebola or respiratory infection*).ti,ab,kw.

6. 1 or 2 or 3 or 4 or 5

7. exp disease outbreaks/ or exp epidemics/ or exp Endemic Diseases/

8. (epidemic* or pandemic* or endemic* or disease outbreak*).ti,ab,kw.

9. 7 or 8

10. (public health message* or hygiene communication or health consult*).ti,ab,kw.

11. exp Health Communication/

12. communication/ or persuasive communication/ or public health message/ or public health campaign/

13. ((health* or prosocial* or moral* or utilitarian or norm* or emotion* or hygiene* or persua* or other-focused or other focused or protect* other*) adj3 (messag* or communicat*)).ti,ab,kw.

14. text messaging/ or mass communication/ or mass medium/ or social media/

15. (mass media or leaflet* or poster* or message framing or text messag* or SMS or social media).ti,ab,kw.

16. 10 or 11 or 12 or 13 or 14 or 15

17. social behavior/ or altruism/ or social distance/ or social isolation/

18. Empathy/

19. Masks/

20. (face covering* or mask* or hand hygiene or hand wash* or social distanc* or social isolation).ti,ab,kw.

21. exp Hand Hygiene/

22. (prosocial* behavior* or pro-social* behaviour* or prosociality or pro-sociality or moral behaviour* or moral behavior* or utilitarian behaviour* or utilitarian behavior* or altruism or empathy).ti,ab,kw.

23. (famil* or community or father* or mother*or parent* or home* or friend* or loved one* or elder* or vulnerable* or other*).ti,ab,kw.

24. Health Risk Behaviors/

25. ((behaviour* or behavio?r*) adj5 (change or influenc* or alter* or modif*)).ti,ab,kw.

26. 17 or 18 or 19 or 20 or 21 or 22 or 23 or 24 or 25

27. 6 and 9 and 16 and 26

28. limit 27 to english language
