# Supplementary material for: Effect of prosocial public health messages for population behaviour change in relation to respiratory infections: a systematic review protocol
Source: BMJ Open. 2021 Jan 13;11(1):e044763. doi: 10.1136/bmjopen-2020-044763 (PMC7812082; doi:10.1136/bmjopen-2020-044763)
Supplement: Author's manuscript [file bmjopen-2020-044763.draft_revisions.pdf]

BMJ Open is committed to open peer review. As part of this commitment we make the peer review history of every article we publish publicly available.

When an article is published we post the peer reviewers' comments and the authors' responses online. We also post the versions of the paper that were used during peer review. These are the versions that the peer review comments apply to.

The versions of the paper that follow are the versions that were submitted during the peer review process. They are not the versions of record or the final published versions. They should not be cited or distributed as the published version of this manuscript.

BMJ Open is an open access journal and the full, final, typeset and author-corrected version of record of the manuscript is available on our site with no access controls, subscription charges or pay-per-view fees (http://bmjopen.bmj.com).

If you have any questions on BMJ Open's open peer review process please email info.bmjopen@bmj.com

BMJ Open

The effect of communication strategies for population behaviour change in relation to respiratory infections: A Systematic Review protocol

Journal:	*BMJ Open*
Manuscript ID	bmjopen-2020-044763
Article Type:	Protocol
Date Submitted by the Author:	12-Sep-2020
Complete List of Authors:	Grimani, Aikaterini; Newcastle University, NIHR Policy Research Unit in Behavioural Science; University of Warwick, Warwick Business School<br>Bonell, Chris; Newcastle University, NIHR Policy Research Unit in Behavioural Science; London School of Hygiene & Tropical Medicine, Public Health and Policy<br>Michie, Susan; Newcastle University, NIHR Policy Research Unit in Behavioural Science; University College London, UCL Psychology and Language Sciences<br>Antonopoulou, Vivi; Newcastle University, NIHR Policy Research Unit in Behavioural Science; University College London, UCL Psychology and Language Sciences<br>Kelly, Mike; Newcastle University, NIHR Policy Research Unit in Behavioural Science; University of Cambridge, School of Clinical Medicine<br>Vlaev, Ivo; Newcastle University, NIHR Policy Research Unit in Behavioural Science; University of Warwick, Warwick Business School
Keywords:	COVID-19, Respiratory infections < THORACIC MEDICINE, Public health < INFECTIOUS DISEASES

SCHOLARONE™ Manuscripts

I, the Submitting Author has the right to grant and does grant on behalf of all authors of the Work (as defined in the below author licence), an exclusive licence and/or a non-exclusive licence for contributions from authors who are: i) UK Crown employees; ii) where BMJ has agreed a CC-BY licence shall apply, and/or iii) in accordance with the terms applicable for US Federal Government officers or employees acting as part of their official duties; on a worldwide, perpetual, irrevocable, royalty-free basis to BMJ Publishing Group Ltd ("BMJ") its licensees and where the relevant Journal is co-owned by BMJ to the co-owners of the Journal, to publish the Work in this journal and any other BMJ products and to exploit all rights, as set out in our licence.

The Submitting Author accepts and understands that any supply made under these terms is made by BMJ to the Submitting Author unless you are acting as an employee on behalf of your employer or a postgraduate student of an affiliated institution which is paying any applicable article publishing charge ("APC") for Open Access articles. Where the Submitting Author wishes to make the Work available on an Open Access basis (and intends to pay the relevant APC), the terms of reuse of such Open Access shall be governed by a Creative Commons licence – details of these licences and which Creative Commons licence will apply to this Work are set out in our licence referred to above.

Other than as permitted in any relevant BMJ Author's Self Archiving Policies, I confirm this Work has not been accepted for publication elsewhere, is not being considered for publication elsewhere and does not duplicate material already published. I confirm all authors consent to publication of this Work and authorise the granting of this licence.

For peer review only

The effect of communication strategies for population behaviour change in relation to respiratory infections: A Systematic Review protocol

Aikaterini Grimani[1, 2], Chris Bonell[1, 3], Susan Michie[1, 4], Vivi Antonopoulou[1, 4], Michael P Kelly,[1, 5], Ivo Vlaev[1, 2]

Correspondence to: Dr Aikaterini Grimani; aikaterini.grimani@wbs.ac.uk
University of Warwick, Warwick Business School, Scarman Rd, CV4 7AL, Coventry, UK

1. NIHR Policy Research Unit in Behavioural Science, Newcastle University, Newcastle, UK
2. Warwick Business School, University of Warwick, Coventry, UK
3. London School of Hygiene & Tropical Medicine, London, UK
4. University College London, UCL Psychology and Language Sciences, London, UK
5. University of Cambridge, School of Clinical Medicine, Cambridge, UK

Key terms communication strategies; protect others; respiratory infections; COVID-19; behavioural change; systematic review

Words: 3068

For peer review only

ABSTRACT

Introduction: The COVID-19 pandemic represents a great societal challenge that requires large-scale behaviour change, widespread collective action and cooperation. Existing literature indicates that several messaging approaches may be effective, including emphasizing the benefits to the recipient, aligning with the recipient's moral values, and focusing on protecting others. Current research indicates that prosocial public health messages that highlight behaviours linked to societal benefits (e.g., protect "each other"), rather than focusing on behaviours that protect oneself (e.g., protect "yourself"), may be a more effective method for communicating strategies related to infectious diseases. To investigate this we will conduct a systematic review that will identify what messages and behaviour change techniques have the potential to optimise the effect on population behaviour in relation to reducing transmission of respiratory infections.

Methods and analysis: A systematic literature search of published and unpublished studies (including grey literature) in electronic databases will be conducted to identify studies that meet our inclusion criteria. The search will be run in four electronic databases: MEDLINE, EMBASE, PsycINFO, and Scopus. We will also conduct supplementary searches in databases of "grey" literature such as PsycEXTRA and Public Health England list of index and alternative sources and resources. A systematic approach to searching, screening, reviewing and data extraction will be applied based on Preferred Reporting Items for Systematic reviews and Meta-Analysis (PRISMA). Titles, abstracts, full-texts for eligibility will be examined independently by researchers. The quality of the included studies will be assessed using the Cochrane risk of bias tool and the risk of bias in non-randomised studies - of Interventions tool. Disagreements will be resolved by a consensus procedure.

Ethics and dissemination: This protocol has been registered with PROSPERO. No ethical approval is required, as there will be no collection of primary data. The synthesized findings will be disseminated through peer-reviewed publication.

Systematic review registration: PROSPERO CRD42020198874

For peer review only - http://bmjopen.bmj.com/site/about/guidelines.xhtml

Strengths and limitations of this study

- The analysis, which will be carried out using the Behaviour Change Wheel framework, will identify the key behaviours and, importantly, drivers for behaviours that may be amenable to change.
- The review will include narratively synthesised sections such as Target Behaviour, Intervention, Context, Mechanisms of Action, Outcome and Effect.
- The inclusion of grey literature will broaden this study in terms of included information.
- A limitations of this review will be the exclusion of papers reported in languages other than English.

For peer review only

INTRODUCTION

The COVID-19 pandemic represents a massive global health crisis which requires the rapid adoption of population protective and physical social distancing behaviours[1]. To prevent infectious disease transmission in a pandemic effective strategies are required to communicate important health messages in a concise and meaningful way that makes it easy for citizens to change behaviour, avoiding community ambivalence and panic[2 3]. Several messaging approaches may be effective, including emphasizing the benefits to the recipient, focusing on protecting others (e.g., 'wash your hands to protect your parents and grandparents'; 'coughing or sneezing into your elbow to protect other people'), aligning with the recipient's moral values, appealing to social consensus or scientific norms and/or highlighting social group approval[4]. Furthermore, enlisting trusted voices has been shown to make public health messages more effective in changing behaviour during epidemics[4]. Abu-Akel, et al. [5] suggested that different spokespersons may be needed for the younger and the older populations, and for rural and urban populations. They found that a government official was more effective than a celebrity actor, particularly in response to current compliance with social distancing measures, and was considered substantially stronger by older respondents despite them having lower risk perception compared to younger respondents. Current findings also suggest that the thought of infecting vulnerable people or large numbers of people can motivate social distancing[6 7]. It may therefore be useful to identify which messages work best on which populations, in order to generate policy support and to ensure individuals' actions help to combat infectious disease transmission.

A group of behavioural and social scientists, who have shared their advice with UK government on COVID-19, have collaborated to develop a series of principles to inform

For peer review only - http://bmjopen.bmj.com/site/about/guidelines.xhtml

interventions to promote whole population adherence to social distancing measures. The key principles are: 1) 'Protect yourself' messages will have limited impact for the general public; 2) Clear and specific guidance; 3) Avoid messages based on fear/disgust in relation to other people; 4)'Protect each other' messages are promising (particularly when building on messages promoting collective identity and supportive social norms); 5) 'Stand together' messages emphasise how our sense of self is rooted in our valued membership of collectivities such as families, neighbourhoods, communities and nation, linked to sense of duty, solidarity and inclusion; 6) 'This is who we are' messages should draw upon the social norms (informal rules that govern behaviour) of groups; 7) 'Make a plan and review it regularly' messages can build on points 2-6, rooted in the psychology of reflective decisions to break emotion- or habit-driven behaviour and help people anticipate possible barriers and enablers to adherence and address them in advance; 8) 'Make it possible' messages are more likely to enable adherence than are messages invoking punishment or castigation, which can provoke unpredictable negative reactions; 9) Avoid authorisation messages; 10) Style of messaging; 11) Theory of change; 12) Co-design[8].

These principles were informed by members' expertise and knowledge of existing theory and evidence, rather than by any formal review of the literature. Webster, et al. [9] rapid review of evidence on how to improve adherence with quarantine, supports some of the principles mentioned above. These include: providing a timely, clear rationale for quarantine, providing clear information about quarantine protocol, emphasising social norms to encourage the behaviour as altruistic, highlighting the benefit that engaging in quarantine will have for public health, emphasising its importance (in particular to those at heightened risk of the disease), and ensuring sufficient supplies are provided, providing assistance for those financially affected by quarantine.

Existing literature illustrates that prosocial public health messages that highlight behaviours related to societal and communal benefits (e.g., protect each other), rather than focusing on behaviours that only benefit the self (e.g., protect yourself), may be an especially effective method[10 11] for communicating public health recommendations related to infectious diseases[12]. Prosocial behaviour is defined as behaviour that benefits others, whether or not it involves an overall cost to self, and includes a variety of important social behaviours such as helping, sharing, and cooperation[13 14]. The term "others" refers to specific individuals or groups of people, and in particular loved ones, vulnerable members (with weakened immune systems such as elderly and chronically ill), health care professionals, co-workers, workers in retail and public services, members of the public, and therefore the society as a whole.

For peer review only - http://bmjopen.bmj.com/site/about/guidelines.xhtml

'Protect each other' messages stress how desired behaviours benefit the group and protect its most vulnerable members, including those we love. For example, young people are responsible for the majority of transmissions of influenza [15], but the majority of deaths happen over 65 years of age[16]. Such messages are usually enhanced by concrete examples, powerful images and the actual voices of those we need to protect, linked to clear, specific advice on how to implement required behaviours[17]. 'Protect each other' messages are promising, particularly when building on messages promoting collective identity and supportive social norms. Messages promoting care for others are rooted in the psychology of social identity[18] social influence[19] and moral behaviour[20], with evidence of benefits in the COVID-19[21] and other health contexts[22]. Such messages sometimes use images and accounts of widespread population adherence (rather than examples of non-adherence) in order to persuade 'conditional co-operators' (those whose willingness to help others is conditional on others doing so) to over-ride individual self-interest and to act in the collective interest[23].

Given the above, we will conduct a systematic review that will identify what messages and behaviour change techniques have the potential to optimise the effect on population behaviour in relation to reducing transmission of respiratory infections. The evidence will be gathered from infectious diseases field across a range of behaviours (such as hand hygiene, social distancing, face masks, touching face, catching coughs/sneezes, testing, reporting contacts, self-isolating if infected), and with a specific focus on populations where a protect-others-message is important i.e., those already infected and those at low risk of serious consequences of infection (young people in influenza or corona virus epidemics).

Objective

The aim of this research is to conduct a systematic review of effective communication strategies for population behaviour change in relation to infectious diseases and to synthesize the evidence on supporting or/ and refine the 'protect each other' principle.

Research questions:

- Are messages focusing on protecting others effective in changing a defined list of behavioural outcomes compared to other messages/ controls?
- What behaviours (e.g., social distancing, hand washing, face touching, using hygiene products etc.) do messages about protecting others appear to affect positively?
- What populations do messages about protecting others appear to affect positively?

For peer review only - http://bmjopen.bmj.com/site/about/guidelines.xhtml

METHODS AND ANALYSIS

The systematic review will be performed following the Preferred Reporting Items for Systematic Reviews and Meta-Analyses (PRISMA) statement.[24 25] The protocol was registered in the International Prospective Register of Systematic Reviews (number CRD42020198874).[26]

A comprehensive literature search of published and unpublished studies ("grey literature") in electronic databases will be conducted. The following electronic databases will be searched from inception to August, 2020: Medline, EMBASE.com, PsycINFO, and Scopus. For unpublished studies we will conduct searches in databases of "grey" literature such as PsycEXTRA and Public Health England list of index and alternative sources and resources. We will also conduct supplementary searches in Google Scholar, hand search relevant journals, and conduct backward and forward citation searching of included studies and relevant reviews.

A search strategy (supplementary Table 1) following PICOS will be adapted (population, intervention, comparison, outcome and study design), including Medical Subject Headings (MeSH) terms and relevant key words[27 28]. The PICOS process facilitates a more evidence-based approach to literature searching and helps to rapidly and accurately locate the best available scientific information, limiting unnecessary searching[28].

Eligibility criteria

Only studies written in the English language will be considered. The specific inclusion criteria of the study will be the following:

- Types of studies to be included: We will include published and unpublished (grey literature) RCTs, quasi experimental studies, or time series analyses.
- Population: We will include the general public including population with or without vulnerabilities and/or people who are/are not already infected. No restrictions placed on geographical region.
- Interventions: We will be looking for interventions focused on communication via mass media, social media or small media (such as leaflets and posters) or health professional advices via consultation where messages about protecting others are included.
- Comparisons: Relevant comparisons will include no message or active control with messages focused on self-protection.
- Primary Outcomes: We will include any behaviour relevant to reducing transmission of respiratory infections (e.g., socializing, hand hygiene, social distancing, face masks, touching face, catching coughs/sneezes, testing, reporting contacts, isolating if infected).

For peer review only - http://bmjopen.bmj.com/site/about/guidelines.xhtml

Patient and public involvement (PPI)

A core component of PRU (Policy Research Unit) policy is to maximise public involvement in all our research activities.[29] Thus, the PRU PPI panel has commented on the protocol and will receive regular updates on the review and comment on outputs.

Selection of studies

All titles and abstracts retrieved by electronic searching will be downloaded to the reference manager database End Note and uploaded to Covidence, one of Cochrane's recommended tools. This web-based software platform has been designed to support more efficient management of systematic reviews and can be used from the beginning of title/abstract screening through the beginning of meta-analysis.[30] Duplicates will be removed and double-screening will be done on a proportion of the retrieved citations until the appropriate agreement (>95%) is achieved. The remaining studies will be screened by one reviewer. The abstracts will be included if they meet the inclusion criteria. The same procedure will be applied to determine the eligibility of studies on the basis of a review of the full texts. Differences in judgment will be resolved through discussion and inclusion of a third rater, if required. The selection process will be recorded and the Preferred Reporting Items for Systematic Reviews and Meta-Analyses flow diagram will be comlpeted.[24]

Data extraction

The process of data extraction will involve one or two reviewers who will generate a data extraction form. The theoretical underpinnings of intervention content will also be considered. The data extraction form, which will be reviewed and refined by the reviewers, will include variables as follows:

- the communication message
- characteristics of the recipient(s) of the communication (protect-others-message)
- characteristics of the "others" who will be protected due to the message
- the manner in which the appeal to protect others is made
- intervention features including
  - MINDSPACE checklist of behavioural economic techniques: the nine most robust effects on behaviour (Messenger, Incentives, Norms, Defaults, Salience, Priming, Affect, Commitment and Ego).[31 32]
  - Behaviour Change Techniques (BCTs) (e.g., observable and replicable components designed to change behaviour)[33 34], intervention functions (education, persuasion,

For peer review only - http://bmjopen.bmj.com/site/about/guidelines.xhtml

incentivisation, coercion, training, restriction, environmental restructuring, modelling, enablement) and policy categories (communication/ marketing, guidelines, fiscal, regulation, legislation, environmental/ social planning, service provision), which are all part of the Behaviour Change Wheel framework.[34 35]

- Primary outcome(s): Any behaviour relevant to reducing transmission of respiratory infections. Where more than one reported outcome is provided, we will use the Grades of Recommendation, Assessment, Development and Evaluation (GRADE) approach to assess the certainty of evidence (separated into those that are critical, important and not important).[36] Outcomes measured at multiple time points will be categorized as following: immediate (within 2 weeks of the intervention delivery), short-term (2–13 weeks after intervention delivery), medium-term (14–50 weeks after intervention delivery) and long-term effects (51 or more weeks after intervention delivery). We will present multiple time points only for critical outcomes. The downgrading of the quality of a body of evidence for a specific outcome will be based on five factors: limitations of study; indirectness of evidence; inconsistency of results; precision of results; and publication bias.
- Results.

Quality assessment

An assessment of the methodological quality of included studies will be conducted using the Cochrane Collaboration Risk of Bias Tool (CCRBT)[37] and the Risk Of Bias In Non-randomised Studies - of Interventions (ROBINS-I)[38 39]. CCRBT is a two-part tool, addressing seven evidence-based domains, namely random sequence generation (selection bias), allocation concealment (selection bias), blinding of participants and personnel (performance bias), blinding of outcome assessment (detection bias), incomplete outcome data (attrition bias), selective outcome reporting (reporting bias) and other sources of bias (other bias). The first part of the tool describes a sufficiently detailed support for judgment about the risk of bias, ensuring its transparency. The second part assigns a judgment relating to the risk of bias for each domain. This is achieved by assigning a judgment of "Low risk" of bias (+), "High risk" of bias (-), or "Unclear risk" of bias (?). According to the Cochrane Collaboration's recommendations, the studies, whose all domains will be rated positively, will be judged as low risk of bias, while the studies with one or more unclear domains will be judged as having unclear risk of bias. Furthermore, the studies, where one or more domains will be rated negatively, will be judged as high risk of bias[40 41].

For peer review only - http://bmjopen.bmj.com/site/about/guidelines.xhtml

ROBINS-I is mainly distinct from CCRBT because of the randomization. The first two domains, covering confounding and selection of participants into the study, address issues before the start of the interventions that are to be compared ("baseline"). The third domain addresses classification of the interventions themselves. The other four domains address issues after the start of interventions: biases due to deviations from intended interventions, missing data, measurement of outcomes, and selection of the reported result. The categories for risk of bias judgements are "Low risk", "Moderate risk", "Serious risk" and "Critical risk" of bias. If none of the answers to the signalling questions for a domain suggests a potential problem then risk of bias for the domain can be judged to be low. Otherwise, potential for bias exists[38]. Two review authors will independently evaluate the methodological quality of each included study using the assessment tools. Discrepancies will be resolved through a consensus procedure.

Data analysis and synthesis

We will compare the communication strategies where messages focus on protecting others against an alternative condition. Randomized trials and quasi-experimental studies will be analysed to develop a taxonomy of interventions where possible describing these in terms of behaviour change techniques and, when appropriate, meta- analytically. In particular, the review will include narratively synthesised sections such as:

- Target Behaviour: What is the desired response? This step involves clearly defining the behavioural response required by the recipient(s) of the communication.
- Intervention: A product, service, activity or structural change, intended to achieve behaviour change. BCTs will be double-coded by trained coders using the Behaviour Change Techniques Taxonomy v1[34] using a BCT extraction form. BCTs will be coded separately for intervention and control groups. The MINDSPACE checklist[31] will also be used as it adds techniques predominantly used in the behavioural economics literature. The reliability of coding of BCTs and MINDSPACE checklist will be assessed using the prevalence and bias-adjusted kappa (PABAK) statistic[42]. PABAK will be used because it adjusts for shared bias in the coders' use of categories and high prevalence of negative agreement (e.g., when both coders agree that codes are absent). We will calculate pooled effect sizes within each pairwise comparison (e.g. intervention type versus control), accounting for the extent of heterogeneity among the studies. If an indication of substantial heterogeneity is determined that cannot be explained through meta-regressions, then we will not produce a pooled estimate and will present only the narrative summary. If we consider that we have high levels of unexplained statistical heterogeneity in any of our

For peer review only - http://bmjopen.bmj.com/site/about/guidelines.xhtml

study groupings, we will investigate this further using subgroup and sensitivity analyses. As is appropriate, a random-effects meta-analysis with the extracted BCTs will be conducted. The meta-analysis will be conducted when a group of studies will be sufficiently homogeneous in terms of subjects involved, interventions, and outcomes to provide a meaningful summary. Although a meta-analysis can be conducted with minimum 2 studies, Valentine, et al. [43] suggest that the combination of very few studies with heterogeneous characteristics makes any kind of synthesis untenable in most cases, while parameter estimation (e.g., the random effects variance component) will likely be poor, rendering conclusions that are highly uncertain.

- Context: attributes of the target population (characteristics of the individuals, groups, sub-populations or populations) and the intervention setting (e.g., social and physical environment).
- Mechanisms of Action (MoA): processes by which intervention influences the target behaviour (moderator factors)[44 45]. The 26 MoA, which are defined as the processes through which a BCT affects behaviour, are taken from the 12 most frequently occurring mechanisms derived from a set of 83 behaviour change theories[46] and the 14 theoretical domains as described in the Theoretical Domains Framework (TDF: Knowledge; Skills; Social/professional role and identity; Beliefs about capabilities; Optimism; Beliefs about consequences; Reinforcement; Intentions; Goals; Memory, attention and decision processes; Environmental context and resources; Social influences; Emotions; and Behavioural regulation)[47]. The TDF, which is developed by synthesising 33 psychological theories aimed at behaviour change, enables interventionists better understand the psychological mechanisms of change [48 49].
- Outcome: the type of the target behaviour in the given scenario (e.g., how and when it is assessed).
- Effect: an estimate of the comparison between the outcomes in the evaluated scenarios.

We will also focus on implications for intervention research. We will assess whether interventions addressing the various outcomes, or some subsets of these, appear to have mediating factors or mechanisms.

Conclusions

We will base our conclusions on findings from the narrative synthesis of included studies for this review. Reducing social interactions with others, has the potential to prevent infection

For peer review only - http://bmjopen.bmj.com/site/about/guidelines.xhtml

during the COVID-19 pandemic. This review will identify potential communication strategies to encourage compliance with social distancing and other protective behaviours. Such insights are important for Government health agencies which will benefit from the evidence regarding effective communication strategies as a promising instrument to prevent infectious disease transmission.

ETHICS AND DISSEMINATION

Ethics approval is not required for this knowledge synthesis. This protocol had been registered with the PROSPERO: International prospective register of systematic reviews. The synthesized findings will be disseminated through peer-reviewed publication. This knowledge synthesis can serve as a guide for effective communication methods used to promote adherence to behaviours that prevent infectious disease transmission during pandemics (such as COVID-19), epidemics or endemics for National Health System (NHS) and Department of Health and Social Care (DHSC) stakeholders and other stakeholders. Reaching people with a message is one thing, influencing and changing their behaviour is quite another. Our behavioural science review will provide tried and tested methods that help us design communication strategies that more effectively influence population behaviour relevant to reducing transmission of respiratory infections.

For peer review only

Author Contributions: AG is the corresponding author and the primary author of the study, conceived the study, contributed to the development of the search strategy, developed the inclusion and exclusion criteria and data extraction criteria and was involved in the conceptualization of the research questions. **CB** conceived the study, contributed to the development of the selection criteria and data extraction criteria, was involved in the conceptualization of the research questions and provided written feedback on the manuscript. **SM** conceived the study and provided written feedback on the manuscript. **VA** contributed to the development of the search strategy and provided written feedback on the manuscript. **MK** contributed to the development of the selection criteria and data extraction criteria and provided written feedback on the manuscript. **IV** conceived the study, contributed to the development of the selection criteria and data extraction criteria, was involved in the conceptualization of the research question and revised the manuscript critically and contributed to it intellectually. All authors have read and approved the final version of the manuscript.

Funding statement:

This study/project is funded by the National Institute for Health Research (NIHR) [Policy Research Unit in Behavioural Science (project reference PR-PRU-1217-20501)]. The views expressed are those of the author(s) and not necessarily those of the NIHR or the Department of Health and Social Care.

Competing interests statement: None declared.

For peer review only - http://bmjopen.bmj.com/site/about/guidelines.xhtml

REFERENCES

1. Prem K, Liu Y, Russell TW, et al. The effect of control strategies to reduce social mixing on outcomes of the COVID-19 epidemic in Wuhan, China: a modelling study. *The Lancet Public Health* 2020

2. Smith JA, Judd J. COVID-19: Vulnerability and the power of privilege in a pandemic. *Health Promotion Journal of Australia* 2020;31(2):158.

3. Contreras GW, MEP M. Getting ready for the next pandemic COVID-19: Why we need to be more prepared and less scared. *Journal of Emergency Management* 2020;18(2):87-89.

4. Van Bavel JJ, Baicker K, Boggio PS, et al. Using social and behavioural science to support COVID-19 pandemic response. *Nature Human Behaviour* 2020:1-12.

5. Abu-Akel A, Spitz A, West R. Who is listening? Spokesperson Effect on Communicating Social and Physical Distancing Measures During the COVID-19 Pandemic. 2020

6. Lunn PD, Timmons S, Barjaková M, et al. Motivating social distancing during the Covid-19 pandemic: An online experiment: Economic and Social Research Institute (ESRI), 2020.

7. Pfattheicher S, Nockur L, Böhm R, et al. The emotional path to action: Empathy promotes physical distancing during the COVID-19 pandemic. 2020

8. Bonell C, Michie S, Reicher S, et al. Harnessing behavioural science in public health campaigns to maintain 'social distancing'in response to the COVID-19 pandemic: key principles. *Epidemiol Community Health* 2020

9. Webster RK, Brooks SK, Smith LE, et al. How to improve adherence with quarantine: Rapid review of the evidence. *Public Health* 2020

10. Kelly BJ, Hornik RC. Effects of framing health messages in terms of benefits to loved ones or others: An experimental study. *Health Communication* 2016;31(10):1284-90.

11. Li M, Taylor EG, Atkins KE, et al. Stimulating influenza vaccination via prosocial motives. *PloS one* 2016;11(7):e0159780.

12. Jordan J, Yoeli E, Rand D. Don't get it or don't spread it? Comparing self-interested versus prosocially framed COVID-19 prevention messaging. 2020

13. Grant AM, Hofmann DA. It's not all about me: motivating hand hygiene among health care professionals by focusing on patients. *Psychological science* 2011;22(12):1494-99.

For peer review only - http://bmjopen.bmj.com/site/about/guidelines.xhtml

14. Penner LA, Dovidio JF, Piliavin JA, et al. Prosocial behavior: Multilevel perspectives. *Annu Rev Psychol* 2005;56:365-92.
15. Thompson M, Shay D, Zhou H, et al. Estimates of deaths associated with seasonal influenza-United States, 1976-2007. *Morbidity Mortality Weekly Report* 2010;59(33):1057-62.
16. Longini Jr IM, Koopman JS, Monto AS, et al. Estimating household and community transmission parameters for influenza. *American journal of epidemiology* 1982;115(5):736-51.
17. Tannenbaum MB, Hepler J, Zimmerman RS, et al. Appealing to fear: A meta-analysis of fear appeal effectiveness and theories. *Psychological bulletin* 2015;141(6):1178.
18. Moran MB, Sussman S. Translating the link between social identity and health behavior into effective health communication strategies: An experimental application using antismoking advertisements. *Health Communication* 2014;29(10):1057-66.
19. Haslam SA, Reicher SD, Platow MJ. The new psychology of leadership: Identity, influence and power: Psychology Press 2010.
20. Haidt J. The righteous mind: Why good people are divided by politics and religion: Vintage 2012.
21. Everett JA, Colombatto C, Chituc V, et al. The effectiveness of moral messages on public health behavioral intentions during the COVID-19 pandemic. 2020
22. Shen L. Targeting smokers with empathy appeal antismoking public service announcements: a field experiment. *Journal of Health Communication* 2015;20(5):573-80.
23. Drury J, Carter H, Cocking C, et al. Facilitating collective resilience in the public in emergencies: Twelve recommendations based on the social identity approach. *Frontiers in public health* 2019;7:141.
24. Moher D, Shamseer L, Clarke M, et al. Preferred reporting items for systematic review and meta-analysis protocols (PRISMA-P) 2015 statement. *Systematic reviews* 2015;4(1):1.
25. Shamseer L, Moher D, Clarke M, et al. Preferred reporting items for systematic review and meta-analysis protocols (PRISMA-P) 2015: elaboration and explanation. *Bmj* 2015;349
26. Grimani A, Bonell C, Michie S, et al. The effect of communication strategies for population behaviour change in relation to infectious disease: a systematic review: PROSPERO 2020 CRD42020198874; [Available from: https://www.crd.york.ac.uk/prospero/display_record.php?ID=CRD42020198874.

For peer review only - http://bmjopen.bmj.com/site/about/guidelines.xhtml

27. Huang M, Névéol A, Lu Z. Recommending MeSH terms for annotating biomedical articles. *Journal of the American Medical Informatics Association* 2011;18(5):660-67.

28. Methley AM, Campbell S, Chew-Graham C, et al. PICO, PICOS and SPIDER: a comparison study of specificity and sensitivity in three search tools for qualitative systematic reviews. *BMC health services research* 2014;14(1):579.

29. PRU Behavioural Science. Partnership Working: Patient and Public Engagement 2020 [Available from: https://research.ncl.ac.uk/behscipru/partnership%20working/.

30. Covidence: Cochrane Community; [Available from: https://community.cochrane.org/help/tools-and-software/covidence accessed 20/05/2020.

31. Dolan P, Hallsworth M, Halpern D, et al. Influencing behaviour: The mindspace way. *Journal of Economic Psychology* 2012;33(1):264-77.

32. Vlaev I, King D, Dolan P, et al. The theory and practice of "nudging": changing health behaviors. *Public Administration Review* 2016;76(4):550-61.

33. Michie S, Wood CE, Johnston M, et al. Behaviour change techniques: the development and evaluation of a taxonomic method for reporting and describing behaviour change interventions (a suite of five studies involving consensus methods, randomised controlled trials and analysis of qualitative data). *Health Technology Assessment* 2015;19(99)

34. Michie S, Richardson M, Johnston M, et al. The behavior change technique taxonomy (v1) of 93 hierarchically clustered techniques: building an international consensus for the reporting of behavior change interventions. *Annals of behavioral medicine* 2013;46(1):81-95.

35. Michie S, Van Stralen MM, West R. The behaviour change wheel: a new method for characterising and designing behaviour change interventions. *Implementation science* 2011;6(1):42.

36. Schünemann HJ, Higgins JP, Vist GE, et al. Completing 'Summary of findings' tables and grading the certainty of the evidence. In: Higgins JPT, Thomas J, Chandler J, et al., eds. Cochrane Handbook for Systematic Reviews of Interventions: The Cochrane Collaboration 2019:375-402.

37. Higgins JP, Altman DG, Gøtzsche PC, et al. The Cochrane Collaboration's tool for assessing risk of bias in randomised trials. *Bmj* 2011;343:d5928.

38. Sterne JA, Hernán MA, Reeves BC, et al. ROBINS-I: a tool for assessing risk of bias in non-randomised studies of interventions. *bmj* 2016;355

For peer review only - http://bmjopen.bmj.com/site/about/guidelines.xhtml

39. Schünemann HJ, Cuello C, Akl EA, et al. GRADE guidelines: 18. How ROBINS-I and other tools to assess risk of bias in nonrandomized studies should be used to rate the certainty of a body of evidence. *Journal of clinical epidemiology* 2019;111:105-14.
40. Higgins JP, Thomas J, Chandler J, et al. Cochrane handbook for systematic reviews of interventions: John Wiley & Sons, 2019.
41. Deeks JJ, Higgins JP, Altman DG. Analysing data and undertaking meta-analyses. *Cochrane handbook for systematic reviews of interventions* 2019:241-84.
42. Michie S, Wood CE, Johnston M, et al. Training to code intervention descriptions using Behaviour Change Technique Taxonomy version 1 (study 3). Behaviour change techniques: the development and evaluation of a taxonomic method for reporting and describing behaviour change interventions (a suite of five studies involving consensus methods, randomised controlled trials and analysis of qualitative data): NIHR Journals Library 2015.
43. Valentine JC, Pigott TD, Rothstein HR. How many studies do you need? A primer on statistical power for meta-analysis. *Journal of Educational Behavioral Statistics* 2010;35(2):215-47.
44. Michie S, Carey RN, Johnston M, et al. From theory-inspired to theory-based interventions: A protocol for developing and testing a methodology for linking behaviour change techniques to theoretical mechanisms of action. *Annals of Behavioral Medicine* 2018;52(6):501-12.
45. Carey RN, Connell LE, Johnston M, et al. Behavior change techniques and their mechanisms of action: a synthesis of links described in published intervention literature. *Annals of Behavioral Medicine* 2019;53(8):693-707.
46. Michie S, West R, Campbell R, et al. ABC of Behaviour Change Theories. London: Silverback Publishing 2014.
47. Cane J, O'Connor D, Michie S. Validation of the theoretical domains framework for use in behaviour change and implementation research. *Implementation science* 2012;7(1):37.
48. Michie S, Atkins L, West R. The behaviour change wheel: a guide to designing interventions. . Great Britain Silverback Publishing 2014.
49. Atkins L, Francis J, Islam R, et al. A guide to using the Theoretical Domains Framework of behaviour change to investigate implementation problems. *Implementation Science* 2017;12(1):77.

For peer review only

For peer review only - http://bmjopen.bmj.com/site/about/guidelines.xhtml

Supplementary Table 1. Sample search strategy terms (Medline)

1. respiratory infections
2. Pandemics/
3. Epidemics/
4. "pandemic*".ab,sh,ti.
5. "epidemic*".ab,sh,ti.
6. "disease outbreak* ".ab,sh,ti.
7. 1 or 2 or 3 or 4 or 5 or 6
8. exp Persuasive Communication/
9. Communication/
10. "protection motivation theory".ab,sh,ti.
11. Social Identification/
12. Health Knowledge, Attitudes, Collective behavior, Practice/ or Social Behavior/
13. "moral behaviour".ab,sh,ti.
14. Altruism/,Safeguarding, collective behavior, prosocial behavior, pro-sociality
15. 8 or 9 or 10 or 11 or 12 or 13 or 14
16. "behav*".ab,sh,ti.
17. "adheren*".ab,sh,ti.
18. "message acceptance".ab,sh,ti.
19. adhering.ab,sh,ti.
20. Health Behavior/
21. health behavior/ or exp health attitudes/ or exp health behavior measures/ or exp self-referral/
22. 16 or 17 or 18 or 19 or 20 or 21
23. 7 and 15 and 22

For peer review only - http://bmjopen.bmj.com/site/about/guidelines.xhtml

Reporting checklist for protocol of a systematic review.

Based on the PRISMA-P guidelines.

Instructions to authors

Complete this checklist by entering the page numbers from your manuscript where readers will find each of the items listed below.

Your article may not currently address all the items on the checklist. Please modify your text to include the missing information. If you are certain that an item does not apply, please write "n/a" and provide a short explanation.

Upload your completed checklist as an extra file when you submit to a journal.

In your methods section, say that you used the PRISMA-Preporting guidelines, and cite them as:

Moher D, Shamseer L, Clarke M, Ghersi D, Liberati A, Petticrew M, Shekelle P, Stewart LA. Preferred Reporting Items for Systematic Review and Meta-Analysis Protocols (PRISMA-P) 2015 statement. Syst Rev. 2015;4(1):1.

		Reporting Item	Page Number
**Title**			
Identification	#1a	Identify the report as a protocol of a systematic review	1
Update	#1b	If the protocol is for an update of a previous systematic review, identify as such	n/a

For peer review only

**Registration**			
	#2	If registered, provide the name of the registry (such as PROSPERO) and registration number	2
**Authors**			
Contact	#3a	Provide name, institutional affiliation, e-mail address of all protocol authors; provide physical mailing address of corresponding author	1
Contribution	#3b	Describe contributions of protocol authors and identify the guarantor of the review	12
**Amendments**			
	#4	If the protocol represents an amendment of a previously completed or published protocol, identify as such and list changes; otherwise, state plan for documenting important protocol amendments	n/a
**Support**			
Sources	#5a	Indicate sources of financial or other support for the review	12
Sponsor	#5b	Provide name for the review funder and / or sponsor	12
Role of sponsor or funder	#5c	Describe roles of funder(s), sponsor(s), and / or institution(s), if any, in developing the protocol	12
**Introduction**			

For peer review only

Rationale	#6	Describe the rationale for the review in the context of what is already known	3-5
Objectives	#7	Provide an explicit statement of the question(s) the review will address with reference to participants, interventions, comparators, and outcomes (PICO)	5
**Methods**			
Eligibility criteria	#8	Specify the study characteristics (such as PICO, study design, setting, time frame) and report characteristics (such as years considered, language, publication status) to be used as criteria for eligibility for the review	6
Information sources	#9	Describe all intended information sources (such as electronic databases, contact with study authors, trial registers or other grey literature sources) with planned dates of coverage	6
Search strategy	#10	Present draft of search strategy to be used for at least one electronic database, including planned limits, such that it could be repeated	6
Study records - data management	#11a	Describe the mechanism(s) that will be used to manage records and data throughout the review	7
Study records - selection process	#11b	State the process that will be used for selecting studies (such as two independent reviewers) through each phase of the review (that is, screening, eligibility and inclusion in meta-analysis)	7

For peer review only

Study records - data collection process	#11c	Describe planned method of extracting data from reports (such as piloting forms, done independently, in duplicate), any processes for obtaining and confirming data from investigators	7-8
Data items	#12	List and define all variables for which data will be sought (such as PICO items, funding sources), any pre-planned data assumptions and simplifications	8
Outcomes and prioritization	#13	List and define all outcomes for which data will be sought, including prioritization of main and additional outcomes, with rationale	8
Risk of bias in individual studies	#14	Describe anticipated methods for assessing risk of bias of individual studies, including whether this will be done at the outcome or study level, or both; state how this information will be used in data synthesis	8-9
Data synthesis	#15a	Describe criteria under which study data will be quantitatively synthesised	9-10
Data synthesis	#15b	If data are appropriate for quantitative synthesis, describe planned summary measures, methods of handling data and methods of combining data from studies, including any planned exploration of consistency (such as I2, Kendall's τ)	9-10
Data synthesis	#15c	Describe any proposed additional analyses (such as sensitivity or subgroup analyses, meta-regression)	9-10

For peer review only

Data synthesis	#15d	If quantitative synthesis is not appropriate, describe the type of summary planned	9-10
Meta-bias(es)	#16	Specify any planned assessment of meta-bias(es) (such as publication bias across studies, selective reporting within studies)	n/a
Confidence in cumulative evidence	#17	Describe how the strength of the body of evidence will be assessed (such as GRADE)	8

None The PRISMA-P checklist is distributed under the terms of the Creative Commons Attribution License CC-BY 4.0. This checklist can be completed online using https://www.goodreports.org/, a tool made by the EQUATOR Network in collaboration with Penelope.ai

For peer review only

BMJ Open

The effect of prosocial public health messages for population behaviour change in relation to respiratory infections: A Systematic Review protocol

Journal:	*BMJ Open*
Manuscript ID	bmjopen-2020-044763.R1
Article Type:	Protocol
Date Submitted by the Author:	18-Nov-2020
Complete List of Authors:	Grimani, Aikaterini; Newcastle University, NIHR Policy Research Unit in Behavioural Science; University of Warwick, Warwick Business School<br>Bonell, Chris; Newcastle University, NIHR Policy Research Unit in Behavioural Science; London School of Hygiene & Tropical Medicine, Public Health and Policy<br>Michie, Susan; Newcastle University, NIHR Policy Research Unit in Behavioural Science; University College London, UCL Psychology and Language Sciences<br>Antonopoulou, Vivi; Newcastle University, NIHR Policy Research Unit in Behavioural Science; University College London, UCL Psychology and Language Sciences<br>Kelly, Michael; Newcastle University, NIHR Policy Research Unit in Behavioural Science; University of Cambridge, School of Clinical Medicine<br>Vlaev, Ivo; Newcastle University, NIHR Policy Research Unit in Behavioural Science; University of Warwick, Warwick Business School
**Primary Subject Heading**:	Communication
Secondary Subject Heading:	Respiratory medicine, Infectious diseases, Public health
Keywords:	COVID-19, Respiratory infections < THORACIC MEDICINE, Public health < INFECTIOUS DISEASES

SCHOLARONE™
Manuscripts

I, the Submitting Author has the right to grant and does grant on behalf of all authors of the Work (as defined in the below author licence), an exclusive licence and/or a non-exclusive licence for contributions from authors who are: i) UK Crown employees; ii) where BMJ has agreed a CC-BY licence shall apply, and/or iii) in accordance with the terms applicable for US Federal Government officers or employees acting as part of their official duties; on a worldwide, perpetual, irrevocable, royalty-free basis to BMJ Publishing Group Ltd ("BMJ") its licensees and where the relevant Journal is co-owned by BMJ to the co-owners of the Journal, to publish the Work in this journal and any other BMJ products and to exploit all rights, as set out in our licence.

The Submitting Author accepts and understands that any supply made under these terms is made by BMJ to the Submitting Author unless you are acting as an employee on behalf of your employer or a postgraduate student of an affiliated institution which is paying any applicable article publishing charge ("APC") for Open Access articles. Where the Submitting Author wishes to make the Work available on an Open Access basis (and intends to pay the relevant APC), the terms of reuse of such Open Access shall be governed by a Creative Commons licence – details of these licences and which Creative Commons licence will apply to this Work are set out in our licence referred to above.

Other than as permitted in any relevant BMJ Author's Self Archiving Policies, I confirm this Work has not been accepted for publication elsewhere, is not being considered for publication elsewhere and does not duplicate material already published. I confirm all authors consent to publication of this Work and authorise the granting of this licence.

For peer review only

The effect of prosocial public health messages for population behaviour change in relation to respiratory infections: A Systematic Review protocol

Aikaterini Grimani[1, 2], Chris Bonell[1, 3], Susan Michie[1, 4], Vivi Antonopoulou[1, 4], Michael P Kelly,[1, 5], Ivo Vlaev[1, 2]

Correspondence to: Dr Aikaterini Grimani; aikaterini.grimani@wbs.ac.uk
University of Warwick, Warwick Business School, Scarman Rd, CV4 7AL, Coventry, UK

1. NIHR Policy Research Unit in Behavioural Science, Newcastle University, Newcastle, UK
2. Warwick Business School, University of Warwick, Coventry, UK
3. London School of Hygiene & Tropical Medicine, London, UK
4. UCL Psychology and Language Sciences, University College London, London, UK
5. Department of Public Health and Primary Care, University of Cambridge, Cambridge, UK

Key terms communication strategies; protect others; respiratory infections; COVID-19; behavioural change; systematic review

Words: 3265

For peer review only

ABSTRACT

Introduction: The COVID-19 pandemic represents a major societal challenge that requires large-scale behaviour change, widespread collective action and cooperation to reduce viral transmission. Existing literature indicates that several messaging approaches may be effective, including emphasizing the benefits to the recipient, aligning with the recipient's moral values, and focusing on protecting others. Current research suggests that prosocial public health messages that highlight behaviours linked to societal benefits (e.g., protecting "each other"), rather than focusing on behaviours that protect oneself (e.g., protecting "yourself"), may be a more effective method for communicating strategies related to infectious disease. To investigate this we will conduct a systematic review that will identify what messages and behaviour change techniques have the potential to optimise the effect on population behaviour in relation to reducing transmission of respiratory infections.

Methods and analysis: A systematic literature search of published and unpublished studies (including grey literature) in electronic databases will be conducted to identify those that meet our inclusion criteria. The search will be run in four electronic databases: MEDLINE, EMBASE, PsycINFO, and Scopus. We will also conduct supplementary searches in databases of "grey" literature such as PsycEXTRA, Social Science Research Network (SSRN), OSF PREPRINTS and use the Google Scholar search engine. A systematic approach to searching, screening, reviewing and data extraction will be applied based on Preferred Reporting Items for Systematic reviews and Meta-Analysis (PRISMA). Titles, abstracts, full-texts for eligibility will be examined independently by researchers. The quality of the included studies will be assessed using the Cochrane risk of bias tool and the risk of bias in non-randomised studies - of Interventions tool. Disagreements will be resolved by a consensus procedure.

Ethics and dissemination: This protocol has been registered with PROSPERO. No ethical approval is required, as there will be no collection of primary data. The synthesized findings will be disseminated through peer-reviewed publication.

Systematic review registration: PROSPERO CRD42020198874

For peer review only - http://bmjopen.bmj.com/site/about/guidelines.xhtml

Strengths and limitations of this study

- The analysis, which will be carried out using the Behaviour Change Wheel framework, will identify the key behaviours and, importantly, drivers for behaviours that may be amenable to change.
- The review will include narratively synthesised sections such as Target Behaviour, Intervention, Context, Mechanisms of Action, Outcome and Effect.
- The inclusion of grey literature will broaden this study in terms of included information.
- A limitations of this review will be the exclusion of papers reported in languages other than English.

For peer review only

INTRODUCTION

The COVID-19 pandemic represents a massive global health crisis which requires the rapid adoption of population protective and physical social distancing behaviours[1]. To prevent infectious disease transmission in a pandemic effective strategies are required to communicate important health messages in a concise and meaningful way that makes it easy for citizens to change behaviour, and avoiding community ambivalence and panic[2 3]. Several messaging approaches may be effective, including emphasizing the benefits to the recipient, focusing on protecting others (e.g., 'wash your hands to protect your parents and grandparents'; 'coughing or sneezing into your elbow to protect other people'), aligning with the recipient's moral values, appealing to social consensus or scientific norms and/or highlighting social group approval[4]. Existing literature reveals the importance of communications and public messaging during pandemic outbreaks on the effective implementation of, and adherence to, face mask and other non-pharmaceutical interventions[5]. Inconsistent information from various sources prompts individuals to question the credibility of available information, sometimes resulting in fear and denial of the pandemic. Relevant studies have found that when the communication efforts are seen as unreliable, inconsistent, sensationalist or unduly alarmist, doubts are likely to be generated. These doubts are likely to influence public behavioural responses to an emerging respiratory infection outbreak and may also lead to people to disregard future advice[6-8]. Considering that the main function of wearing a mask is to reduce disease transmission, wearers' behaviour could be depicted as altruistic. This protective behaviour regarding wearing a mask could promote social responsibility and solidarity against a common threat. Campaigns in several countries rapidly reshaped societal norms around the acceptability of wearing a mask in public. Social media were used to share messaging about making them at home or to show

celebrity support for the campaign, creating a movement, which prompted others to imitate this behaviour and follow the example. Slogans such as "my mask protects you, your mask protects me" seem to be considered attractive on a set of social norms[9 10].

Enlisting trusted voices has been shown to make public health messages more effective in changing behaviour during epidemics[4 11 12]. Abu-Akel, et al. [13] suggested that different spokespersons may be needed for the younger and the older populations, and for rural and urban populations. They found that a government official was more effective than a celebrity actor, particularly in response to compliance with social distancing measures, and was considered substantially stronger by older respondents despite them having lower risk perception compared to younger respondents. Current findings also suggest that the thought of infecting vulnerable people or large numbers of people can motivate social distancing[11 14-17]. It may therefore be useful to identify which public health messages work best on which populations, in order to generate policy support and to ensure individuals' actions help to reduce infectious disease transmission.

A group of behavioural and social scientists, who have shared their advice with UK government on COVID-19, have collaborated to develop a series of principles to inform interventions to promote whole population adherence to social distancing. The experts resulted to 11 key principles based on their expertise and the knowledge of existing theory and evidence[18]. A promising key principle, which promotes care for others rather than individual self-interest, is based on social identity[19], social influence[20] and moral behaviour[21]. These 'Protect each other' messages highlight the benefits of protective behaviours for the group and its most vulnerable members, including our loved ones, with evidence of benefits in the COVID-19[22] and other health contexts[23]. For example, young people are responsible for the majority of transmissions of influenza [24], but the majority of deaths happen over 65 years of age[25]. Such messages are usually enhanced by concrete examples, powerful images and the actual voices of those we need to protect, linked to clear, specific advice on how to implement desired behaviours[7 18 26]. In order to persuade those who are willing to help others to act in the collective interest, such messages sometimes use images and accounts of widespread population adherence[18 27]. In addition, such messages are expected to be efficacious for those who consider themselves at low risk of severe consequences from COVID-19 infection due to optimism bias[18].

According to the group of experts, the other 10 key principles are:

- Messages should come from representative and trusted personages who can influence and urge diverse groups to help each other;

For peer review only - http://bmjopen.bmj.com/site/about/guidelines.xhtml

- Messages should reflect group culture and behaviour, using social norms;
- Messages should give clear, specific and calm advice, enabling people to plan how to commit to protective behaviours and review these plans regularly;
- Use a theory of change of how campaign activities aim to generate behavioural impacts;
- Avoid messages based on fear/disgust in relation to other people;
- Avoid authoritarian messages, as evidence shows that individuals differ markedly in their receptiveness to what may be seen as authoritarian moral messages;
- Messages which promote reward, incentives and enablement tend to be more effective influences on desired behaviours;
- Messages should be communicated via professionally designed and appealing mass and social media campaigns with trusted spokespeople;
- There is a need for clear and specific guidance on the protective behaviours the general population should adopt;
- The interventions should be co-designed, piloted and evaluated, using a range of methods[18].

Webster, et al. [28] rapid review of the evidence on how to improve adherence with quarantine, supports some of the principles mentioned above. These include: providing a timely, clear rationale for quarantine, providing clear information about quarantine protocol, emphasising social norms to encourage the behaviour as altruistic, highlighting the benefit that engaging in quarantine will have for public health, emphasising its importance (in particular to those at heightened risk of the disease), and ensuring sufficient supplies are provided, providing assistance for those financially affected by quarantine.

The existing literature illustrates that prosocial public health messages that highlight behaviours related to societal and communal benefits (e.g., protect each other), rather than focusing on behaviours that only benefit the self (e.g., protect yourself), may be an especially effective method[29-31] for communicating public health recommendations related to infectious diseases[7 32 33]. Prosocial behaviour is defined as behaviour that benefits others, whether or not it involves an overall cost to self, and includes a variety of important social behaviours such as helping, sharing, and cooperation[34 35]. The term "others" refers to specific individuals or groups of people, and in particular loved ones, vulnerable members (with weakened immune systems such as elderly and chronically ill), health care professionals, co-workers, workers in retail and public services, members of the public, and therefore the society as a whole.

For peer review only - http://bmjopen.bmj.com/site/about/guidelines.xhtml

Given the above, we will conduct a systematic review that will identify what messages and behaviour change techniques have the potential to optimise the effect on population behaviour in relation to reducing transmission of respiratory infections. The evidence will be gathered from infectious diseases field across a range of behaviours (such as hand hygiene, social distancing, face masks, touching face, catching coughs/sneezes, testing, reporting contacts, self-isolating if infected), and with a specific focus on populations where a protect-others-message is important i.e., those already infected and those at low risk of serious consequences of infection (young people in influenza or corona virus epidemics). Such insights are important for Government health agencies which will benefit from the evidence regarding effective communication strategies as a promising instrument to prevent infectious disease transmission.

Objective

The aim of this research is to conduct a systematic review of effective communication strategies for population behaviour change in relation to infectious diseases and to synthesize the evidence on supporting or/ and refining the 'protect each other' principle.

Research questions:

- Are messages focusing on protecting others effective in changing a defined list of behavioural outcomes compared to other messages/ controls?
- What behaviours (e.g., social distancing, hand washing, face touching, using hygiene products etc.) do messages about protecting others appear to affect positively?
- What populations do messages about protecting others appear to affect positively?

METHODS AND ANALYSIS

The systematic review will be performed following the Preferred Reporting Items for Systematic Reviews and Meta-Analyses (PRISMA) statement.[36 37] The protocol was registered in the International Prospective Register of Systematic Reviews (number CRD42020198874).[38]

A comprehensive literature search of published and unpublished studies ("grey literature") in electronic databases will be conducted. The following electronic databases will be searched from inception to October, 2020: Medline, EMBASE.com, PsycINFO, and Scopus. For unpublished studies we will conduct searches in databases of "grey" literature such

For peer review only - http://bmjopen.bmj.com/site/about/guidelines.xhtml

as PsycEXTRA, Social Science Research Network (SSRN), OSF PREPRINTS database which includes BioHackrXiv, Cogprints, MediArXiv, SocArXiv, PsyArXiv and RePEc. We will also conduct supplementary searches in Google Scholar, hand search relevant journals, and conduct backward and forward citation searching of included studies and relevant reviews.

A search strategy (supplementary Table 1) following PICOS will be adapted (population, intervention, comparison, outcome and study design), including Medical Subject Headings (MeSH) terms and relevant key words[39 40]. The PICOS process will facilitate an evidence-based approach to literature searching and help to rapidly and accurately locate the best available scientific information, limiting unnecessary searching[40].

Eligibility criteria

Only studies written in the English language will be considered. The specific inclusion criteria of the study will be the following:

- Types of studies to be included: We will include published and unpublished (grey literature) RCTs, quasi experimental studies, or time series analyses.
- Population: We will include the general public including population with or without vulnerabilities and/or people who are/are not already infected. There will be no restrictions placed on geographical region.
- Interventions: We will be looking for interventions focused on communication via mass media, social media or print media (such as leaflets and posters) or health professional advices via consultation where messages about protecting others are included.
- Comparisons: Relevant comparisons will include no message or active control with messages focused on self-protection or messages that contain no motivational content.
- Primary Outcomes: We will include any behaviour relevant to reducing transmission of respiratory infections (e.g., socializing, hand hygiene, social distancing, face masks, touching face, catching coughs/sneezes, testing, reporting contacts, isolating if infected).

Patient and public involvement (PPI)

A core component of PRU (Policy Research Unit) policy is to maximise public involvement in all our research activities.[41] Thus, the PRU PPI panel has commented on the protocol and will receive regular updates on the review and comment on outputs.

Selection of studies

All titles and abstracts retrieved by electronic searching will be downloaded to the reference manager database End Note and uploaded to Covidence, one of Cochrane's recommended

For peer review only - http://bmjopen.bmj.com/site/about/guidelines.xhtml

tools. This web-based software platform has been designed to support more efficient management of systematic reviews and can be used from the beginning of title/abstract screening through the beginning of meta-analysis.[42] Duplicates will be removed and double-screening will be done on a proportion of the retrieved citations until the appropriate agreement (>95%) is achieved. The remaining studies will be screened by one reviewer. The abstracts will be included if they meet the inclusion criteria. The same procedure will be applied to determine the eligibility of studies on the basis of a review of the full texts. Differences in judgment will be resolved through discussion and inclusion of a third researcher doing the rating, if required. The selection process will be recorded and the Preferred Reporting Items for Systematic Reviews and Meta-Analyses flow diagram will be comlpeted.[36]

Data extraction

The process of data extraction will involve one or two reviewers who will generate a data extraction form. The theoretical underpinnings of intervention content will also be considered. The data extraction form, which will be reviewed and refined by the reviewers, will include variables as follows:

- the communication message
- characteristics of the recipient(s) of the communication (protect-others-message)
- characteristics of the "others" who will be protected due to the message
- the manner in which the appeal to protect others is made
- intervention features including
  - MINDSPACE checklist of behavioural economic techniques: Messenger, Incentives, Norms, Defaults, Salience, Priming, Affect, Commitment and Ego.[43 44]
  - Behaviour Change Techniques (BCTs) (e.g., observable and replicable components designed to change behaviour)[45], intervention functions (education, persuasion, incentivisation, coercion, training, restriction, environmental restructuring, modelling, enablement) and policy categories (communication/ marketing, guidelines, fiscal, regulation, legislation, environmental/ social planning, service provision), which are all part of the Behaviour Change Wheel framework.[46]
  - Primary outcome(s): Any behaviour relevant to reducing transmission of respiratory infections. Where more than one reported outcome is provided, we will use the Grades of Recommendation, Assessment, Development and Evaluation (GRADE) approach to assess the certainty of evidence (separated into those that are critical [any behaviour

For peer review only - http://bmjopen.bmj.com/site/about/guidelines.xhtml

relevant to reducing transmission of respiratory infections] and not important [other outcomes]).[47] Outcomes measured at multiple time points will be categorized as following: immediate (within 2 weeks of the intervention delivery), short-term (2–13 weeks after intervention delivery), medium-term (14–50 weeks after intervention delivery) and long-term effects (51 or more weeks after intervention delivery). We will present multiple time points only for critical outcomes. The downgrading of the quality of a body of evidence for a specific outcome will be based on five factors: limitations of study; indirectness of evidence; inconsistency of results; precision of results; and publication bias.

- Results.

Quality assessment

An assessment of the methodological quality of included studies will be conducted using the Cochrane Collaboration Risk of Bias Tool (RoB 2)[48] and the Risk Of Bias In Non-randomised Studies - of Interventions (ROBINS-I)[49 50]. RoB 2 is structured into five bias domains (bias arising from the randomisation process; bias due to deviations from intended interventions; bias due to missing outcome data; bias in measurement of the outcome; bias in selection of the reported result). Within each domain, the assessment comprises: a series of signalling questions; a judgment about risk of bias for the domain, facilitated by an algorithm that maps responses to signalling questions to a proposed judgment; free text boxes to justify responses to the signalling questions and risk-of-bias judgments; optional free text boxes to predict (and explain) the likely direction of bias. The response options are "yes," "probably yes," "probably no," "no," and "no information." The risk-of-bias judgments for each domain are "low risk of bias," "some concerns," or "high risk of bias." According to the Cochrane Collaboration's recommendations, the studies, whose all domains will be rated low risk, will be judged as low risk of bias, while the studies with one or more concerns will be judged to raise some concerns. Furthermore, the studies, where one or more domains will be rated high risk, will be judged to be at high risk of bias[51].

ROBINS-I is mainly distinct from RoB 2 because of the randomization. The first two domains, covering confounding and selection of participants into the study, address issues before the start of the interventions that are to be compared ("baseline"). The third domain addresses classification of the interventions themselves. The other four domains address issues after the start of interventions: biases due to deviations from intended interventions, missing data, measurement of outcomes, and selection of the reported result. The categories for risk of

For peer review only - http://bmjopen.bmj.com/site/about/guidelines.xhtml

bias judgements are "Low risk", "Moderate risk", "Serious risk" and "Critical risk" of bias. If none of the answers to the signalling questions for a domain suggests a potential problem then risk of bias for the domain can be judged to be low. Otherwise, potential for bias exists[49]. Two review authors will independently evaluate the methodological quality of each included study using the assessment tools. Discrepancies will be resolved through a consensus procedure.

Data analysis and synthesis

We will compare the communication strategies where messages focus on protecting others against an alternative condition. Randomized trials and quasi-experimental studies will be analysed to develop a taxonomy of interventions where possible describing these in terms of behaviour change techniques and, when appropriate, meta- analytically. In particular, the review will include narratively synthesised sections such as:

- Target Behaviour: What is the desired response? This step involves clearly defining the behavioural response required by the recipient(s) of the communication.
- Intervention: A product, service, activity or structural change, intended to achieve behaviour change. BCTs will be double-coded by trained coders using the Behaviour Change Techniques Taxonomy v1[45] using a BCT extraction form. BCTs will be coded separately for intervention and control groups. The MINDSPACE checklist[43] will also be used as it adds techniques predominantly used in the behavioural economics literature. The reliability of coding of BCTs and MINDSPACE checklist will be assessed using the prevalence and bias-adjusted kappa (PABAK) statistic[52]. PABAK will be used because it adjusts for shared bias in the coders' use of categories and high prevalence of negative agreement (e.g., when both coders agree that codes are absent). We will calculate pooled effect sizes within each pairwise comparison (e.g. intervention type versus control), accounting for the extent of heterogeneity among the studies. If an indication of substantial heterogeneity is determined that cannot be explained through meta-regressions, then we will not produce a pooled estimate and will present only the narrative summary. If we consider that we have high levels of unexplained statistical heterogeneity in any of our study groupings, we will investigate this further using subgroup and sensitivity analyses. As is appropriate, a random-effects meta-analysis with the extracted BCTs will be conducted. The meta-analysis will be conducted when a group of studies will be sufficiently homogeneous in terms of subjects involved, interventions, and outcomes to provide a meaningful summary. Although a meta-analysis can be conducted with minimum 2 studies, Valentine, et al. [53] suggest that the combination of very few studies

For peer review only - http://bmjopen.bmj.com/site/about/guidelines.xhtml

with heterogeneous characteristics makes any kind of synthesis untenable in most cases, while parameter estimation (e.g., the random effects variance component) will likely be poor, rendering conclusions that are highly uncertain.

- Context: attributes of the target population (characteristics of the individuals, groups, sub-populations or populations) and the intervention setting (e.g., social and physical environment).
- Mechanisms of Action (MoA): processes by which intervention influences the target behaviour (moderator factors)[54]. The 26 MoA, which are defined as the processes through which a BCT affects behaviour, are taken from the 12 most frequently occurring mechanisms derived from a set of 83 behaviour change theories[46] and the 14 theoretical domains as described in the Theoretical Domains Framework (TDF: Knowledge; Skills; Social/professional role and identity; Beliefs about capabilities; Optimism; Beliefs about consequences; Reinforcement; Intentions; Goals; Memory, attention and decision processes; Environmental context and resources; Social influences; Emotions; and Behavioural regulation)[55]. The TDF, which is developed by synthesising 33 psychological theories aimed at behaviour change, enables interventionists better understand the psychological mechanisms of change [46 56].
- Outcome: the type of the target behaviour in the given scenario (e.g., how and when it is assessed).
- Effect: an estimate of the comparison between the outcomes in the evaluated scenarios.

We will also focus on implications for intervention research. We will assess whether interventions addressing the various outcomes, or some subsets of these, appear to have mediating factors or mechanisms.

ETHICS AND DISSEMINATION

Ethics approval is not required for this knowledge synthesis. This protocol had been registered with the PROSPERO: International prospective register of systematic reviews. The synthesized findings will be disseminated through peer-reviewed publication. This knowledge synthesis can serve as a guide for effective communication methods used to promote adherence to behaviours that prevent infectious disease transmission during pandemics (such as COVID-19), epidemics or endemics for National Health System (NHS) and Department of Health and Social Care (DHSC) stakeholders and other stakeholders. Reaching people with a message is one thing, influencing and changing their behaviour is quite another. Our behavioural science

For peer review only - http://bmjopen.bmj.com/site/about/guidelines.xhtml

For peer review only

review will provide tried and tested methods that help us design communication strategies that more effectively influence population behaviour relevant to reducing transmission of respiratory infections.

For peer review only

For peer review only - http://bmjopen.bmj.com/site/about/guidelines.xhtml

Author Contributions: **AG** is the corresponding author and the primary author of the study, conceived the study, contributed to the development of the search strategy, developed the inclusion and exclusion criteria and data extraction criteria and was involved in the conceptualization of the research questions. **CB** conceived the study, contributed to the development of the selection criteria and data extraction criteria, was involved in the conceptualization of the research questions and provided written feedback on the manuscript. **SM** conceived the study and provided written feedback on the manuscript. **VA** contributed to the development of the search strategy and provided written feedback on the manuscript. **MK** contributed to the development of the selection criteria and data extraction criteria and provided written feedback on the manuscript. **IV** conceived the study, contributed to the development of the selection criteria and data extraction criteria, was involved in the conceptualization of the research question and revised the manuscript critically and contributed to it intellectually. All authors have read and approved the final version of the manuscript.

Funding statement:

This study/project is funded by the National Institute for Health Research (NIHR) [Policy Research Unit in Behavioural Science (project reference PR-PRU-1217-20501)]. The views expressed are those of the author(s) and not necessarily those of the NIHR or the Department of Health and Social Care.

Competing interests statement: None declared.

For peer review only - http://bmjopen.bmj.com/site/about/guidelines.xhtml

REFERENCES

1. Prem K, Liu Y, Russell TW, et al. The effect of control strategies to reduce social mixing on outcomes of the COVID-19 epidemic in Wuhan, China: a modelling study. *The Lancet Public Health* 2020

2. Smith JA, Judd J. COVID-19: Vulnerability and the power of privilege in a pandemic. *Health Promotion Journal of Australia* 2020;31(2):158.

3. Contreras GW, MEP M. Getting ready for the next pandemic COVID-19: Why we need to be more prepared and less scared. *Journal of Emergency Management* 2020;18(2):87-89.

4. Van Bavel JJ, Baicker K, Boggio PS, et al. Using social and behavioural science to support COVID-19 pandemic response. *Nature Human Behaviour* 2020:1-12.

5. Caress A, Duxbury P, Woodcock A, et al. Exploring the needs, concerns and behaviours of people with existing respiratory conditions in relation to the H1N1'swine influenza'pandemic: a multicentre survey and qualitative study. *Health technology assessment* 2010;14(34):1-108.

6. Seale H, Mak JP, Razee H, et al. Examining the knowledge, attitudes and practices of domestic and international university students towards seasonal and pandemic influenza. *BMC Public Health* 2012;12(1):307.

7. Ghio D, Lawes-Wickwar S, Tang MY, et al. What influences people's responses to public health messages for managing risks and preventing disease during public health crises? A rapid review of the evidence and recommendations. 2020

8. Teasdale E, Santer M, Geraghty AW, et al. Public perceptions of non-pharmaceutical interventions for reducing transmission of respiratory infection: systematic review and synthesis of qualitative studies. *BMC Public Health* 2014;14(1):589.

9. van der Westhuizen H-M, Kotze K, Tonkin-Crine S, et al. Face coverings for covid-19: from medical intervention to social practice. *BMJ* 2020;370 doi: https://doi.org/10.1136/bmj.m3021

10. Cheng KK, Lam TH, Leung CC. Wearing face masks in the community during the COVID-19 pandemic: altruism and solidarity. *The Lancet* 2020 doi: https://doi.org/10.1016/S0140-6736(20)30918-1

11. Abu-Akel A, Spitz A, West R. The Fauci Effect: Public Health Messaging during the Covid-19 Pandemic. 2020

For peer review only - http://bmjopen.bmj.com/site/about/guidelines.xhtml

12. Vaughan E, Tinker T. Effective health risk communication about pandemic influenza for vulnerable populations. *American journal of public health* 2009;99(S2):S324-S32.

13. Abu-Akel A, Spitz A, West R. Who is listening? Spokesperson Effect on Communicating Social and Physical Distancing Measures During the COVID-19 Pandemic. 2020

14. Lunn PD, Timmons S, Barjaková M, et al. Motivating social distancing during the Covid-19 pandemic: An online experiment: Economic and Social Research Institute (ESRI), 2020.

15. Nan X. Social distance, framing, and judgment: A construal level perspective. *Human Communication Research* 2007;33(4):489-514.

16. Pfattheicher S, Nockur L, Böhm R, et al. The emotional path to action: Empathy promotes physical distancing during the COVID-19 pandemic. 2020

17. Han Q, Zheng B, Cristea M, et al. Trust in government and its associations with health behaviour and prosocial behaviour during the COVID-19 pandemic. 2020 doi: https://doi.org/10.31234/osf.io/p5gns

18. Bonell C, Michie S, Reicher S, et al. Harnessing behavioural science in public health campaigns to maintain 'social distancing'in response to the COVID-19 pandemic: key principles. *Epidemiol Community Health* 2020;74(8):617-19.

19. Moran MB, Sussman S. Translating the link between social identity and health behavior into effective health communication strategies: An experimental application using antismoking advertisements. *Health Communication* 2014;29(10):1057-66.

20. Haslam SA, Reicher SD, Platow MJ. The new psychology of leadership: Identity, influence and power: Psychology Press 2010.

21. Haidt J. The righteous mind: Why good people are divided by politics and religion: Vintage 2012.

22. Everett JA, Colombatto C, Chituc V, et al. The effectiveness of moral messages on public health behavioral intentions during the COVID-19 pandemic. 2020

23. Shen L. Targeting smokers with empathy appeal antismoking public service announcements: a field experiment. *Journal of Health Communication* 2015;20(5):573-80.

24. Thompson M, Shay D, Zhou H, et al. Estimates of deaths associated with seasonal influenza-United States, 1976-2007. *Morbidity Mortality Weekly Report* 2010;59(33):1057-62.

For peer review only - http://bmjopen.bmj.com/site/about/guidelines.xhtml

25. Longini Jr IM, Koopman JS, Monto AS, et al. Estimating household and community transmission parameters for influenza. *American journal of epidemiology* 1982;115(5):736-51.

26. Tannenbaum MB, Hepler J, Zimmerman RS, et al. Appealing to fear: A meta-analysis of fear appeal effectiveness and theories. *Psychological bulletin* 2015;141(6):1178.

27. Drury J, Carter H, Cocking C, et al. Facilitating collective resilience in the public in emergencies: Twelve recommendations based on the social identity approach. *Frontiers in public health* 2019;7:141.

28. Webster RK, Brooks SK, Smith LE, et al. How to improve adherence with quarantine: Rapid review of the evidence. *Public Health* 2020

29. Kelly BJ, Hornik RC. Effects of framing health messages in terms of benefits to loved ones or others: An experimental study. *Health Communication* 2016;31(10):1284-90.

30. Li M, Taylor EG, Atkins KE, et al. Stimulating influenza vaccination via prosocial motives. *PloS one* 2016;11(7):e0159780.

31. Balbo L, Jeannot F, Estarague J. Combining message framing and social distance to promote pro-social health behaviors. 31ème Congrès de l'Association Française du Marketing-AFM 2015, 2015.

32. Jordan J, Yoeli E, Rand D. Don't get it or don't spread it? Comparing self-interested versus prosocially framed COVID-19 prevention messaging. 2020

33. Curtis V, Dreibelbis R, Sidibe M, et al. How to Set up Government-Led National Hygiene Communication Campaigns to Combat COVID-19: A Strategic Blueprint. *BMJ Global Health* 2020;5:e002780. doi: doi:10.1136/bmjgh-2020-002780

34. Grant AM, Hofmann DA. It's not all about me: motivating hand hygiene among health care professionals by focusing on patients. *Psychological science* 2011;22(12):1494-99.

35. Penner LA, Dovidio JF, Piliavin JA, et al. Prosocial behavior: Multilevel perspectives. *Annu Rev Psychol* 2005;56:365-92.

36. Moher D, Shamseer L, Clarke M, et al. Preferred reporting items for systematic review and meta-analysis protocols (PRISMA-P) 2015 statement. *Systematic reviews* 2015;4(1):1.

37. Shamseer L, Moher D, Clarke M, et al. Preferred reporting items for systematic review and meta-analysis protocols (PRISMA-P) 2015: elaboration and explanation. *Bmj* 2015;349

38. Grimani A, Bonell C, Michie S, et al. The effect of communication strategies for population behaviour change in relation to infectious disease: a systematic review: PROSPERO 2020 CRD42020198874; [Available from: https://www.crd.york.ac.uk/prospero/display_record.php?ID=CRD42020198874.

For peer review only

39. Huang M, Névéol A, Lu Z. Recommending MeSH terms for annotating biomedical articles. *Journal of the American Medical Informatics Association* 2011;18(5):660-67.
40. Methley AM, Campbell S, Chew-Graham C, et al. PICO, PICOS and SPIDER: a comparison study of specificity and sensitivity in three search tools for qualitative systematic reviews. *BMC health services research* 2014;14(1):579.
41. PRU Behavioural Science. Partnership Working: Patient and Public Engagement 2020 [Available from: https://research.ncl.ac.uk/behscipru/partnership%20working/.
42. Covidence: Cochrane Community; [Available from: https://community.cochrane.org/help/tools-and-software/covidence accessed 20/05/2020.
43. Dolan P, Hallsworth M, Halpern D, et al. Influencing behaviour: The mindspace way. *Journal of Economic Psychology* 2012;33(1):264-77.
44. Vlaev I, King D, Dolan P, et al. The theory and practice of "nudging": changing health behaviors. *Public Administration Review* 2016;76(4):550-61.
45. Michie S, Richardson M, Johnston M, et al. The behavior change technique taxonomy (v1) of 93 hierarchically clustered techniques: building an international consensus for the reporting of behavior change interventions. *Annals of behavioral medicine* 2013;46(1):81-95.
46. Michie S, Atkins L, West R. The behaviour change wheel: a guide to designing interventions. Great Britain Silverback Publishing 2014.
47. Schünemann HJ, Higgins JP, Vist GE, et al. Completing 'Summary of findings' tables and grading the certainty of the evidence. In: Higgins JPT, Thomas J, Chandler J, et al., eds. Cochrane Handbook for Systematic Reviews of Interventions: The Cochrane Collaboration 2019:375-402.
48. Sterne JA, Savović J, Page MJ, et al. RoB 2: a revised tool for assessing risk of bias in randomised trials. *BMJ* 2019;366
49. Sterne JA, Hernán MA, Reeves BC, et al. ROBINS-I: a tool for assessing risk of bias in non-randomised studies of interventions. *bmj* 2016;355
50. Schünemann HJ, Cuello C, Akl EA, et al. GRADE guidelines: 18. How ROBINS-I and other tools to assess risk of bias in nonrandomized studies should be used to rate the certainty of a body of evidence. *Journal of clinical epidemiology* 2019;111:105-14.
51. Higgins JP, Thomas J, Chandler J, et al. Cochrane handbook for systematic reviews of interventions. 2nd Edition. Chichester (UK): John Wiley & Sons, 2019.

For peer review only - http://bmjopen.bmj.com/site/about/guidelines.xhtml

52. Michie S, Wood CE, Johnston M, et al. Training to code intervention descriptions using Behaviour Change Technique Taxonomy version 1 (study 3). Behaviour change techniques: the development and evaluation of a taxonomic method for reporting and describing behaviour change interventions (a suite of five studies involving consensus methods, randomised controlled trials and analysis of qualitative data): NIHR Journals Library 2015.

53. Valentine JC, Pigott TD, Rothstein HR. How many studies do you need? A primer on statistical power for meta-analysis. *Journal of Educational Behavioral Statistics* 2010;35(2):215-47.

54. Carey RN, Connell LE, Johnston M, et al. Behavior change techniques and their mechanisms of action: a synthesis of links described in published intervention literature. *Annals of Behavioral Medicine* 2019;53(8):693-707.

55. Cane J, O'Connor D, Michie S. Validation of the theoretical domains framework for use in behaviour change and implementation research. *Implementation science* 2012;7(1):37.

56. Atkins L, Francis J, Islam R, et al. A guide to using the Theoretical Domains Framework of behaviour change to investigate implementation problems. *Implementation Science* 2017;12(1):77.

For peer review only - http://bmjopen.bmj.com/site/about/guidelines.xhtml

Supplementary Table 1. Search strategy terms (Medline)

1. exp respiratory tract infections/

2. exp Coronavirus Infections/ or exp SARS Virus/ or exp Severe Acute Respiratory Syndrome/

3. exp Coronavirus/

4. exp Influenza A Virus, H1N1 Subtype/ or exp Influenza, Human/

5. (covid-19 or coronavir* or corona virus* or covid19 or covid 19 or coronavirus infection* or coronavirus or SARS virus or CoV2 or sarscov2 or Severe Acute Respiratory Syndrome or influenza or flu or H1N1 or Ebola or respiratory infection*).ti,ab,kw.

6. 1 or 2 or 3 or 4 or 5

7. exp disease outbreaks/ or exp epidemics/ or exp Endemic Diseases/

8. (epidemic* or pandemic* or endemic* or disease outbreak*).ti,ab,kw.

9. 7 or 8

10. (public health message* or hygiene communication or health consult*).ti,ab,kw.

11. exp Health Communication/

12. communication/ or persuasive communication/ or public health message/ or public health campaign/

13. ((health* or prosocial* or moral* or utilitarian or norm* or emotion* or hygiene* or persua* or other-focused or other focused or protect* other*) adj3 (messag* or communicat*)).ti,ab,kw.

14. text messaging/ or mass communication/ or mass medium/ or social media/

15. (mass media or leaflet* or poster* or message framing or text messag* or SMS or social media).ti,ab,kw.

16. 10 or 11 or 12 or 13 or 14 or 15

17. social behavior/ or altruism/ or social distance/ or social isolation/

18. Empathy/

19. Masks/

20. (face covering* or mask* or hand hygiene or hand wash* or social distanc* or social isolation).ti,ab,kw.

21. exp Hand Hygiene/

22. (prosocial* behavior* or pro-social* behaviour* or prosociality or pro-sociality or moral behaviour* or moral behavior* or utilitarian behaviour* or utilitarian behavior* or altruism or empathy).ti,ab,kw.

23. (famil* or community or father* or mother*or parent* or home* or friend* or loved one* or elder* or vulnerable* or other*).ti,ab,kw.

24. Health Risk Behaviors/

For peer review only

25. ((behaviour* or behavio?r*) adj5 (change or influenc* or alter* or modif*)).ti,ab,kw.

26. 17 or 18 or 19 or 20 or 21 or 22 or 23 or 24 or 25

27. 6 and 9 and 16 and 26

28. limit 27 to english language

For peer review only

Reporting checklist for protocol of a systematic review.

Based on the PRISMA-P guidelines.

Instructions to authors

Complete this checklist by entering the page numbers from your manuscript where readers will find each of the items listed below.

Your article may not currently address all the items on the checklist. Please modify your text to include the missing information. If you are certain that an item does not apply, please write "n/a" and provide a short explanation.

Upload your completed checklist as an extra file when you submit to a journal.

In your methods section, say that you used the PRISMA-Preporting guidelines, and cite them as:

Moher D, Shamseer L, Clarke M, Ghersi D, Liberati A, Petticrew M, Shekelle P, Stewart LA. Preferred Reporting Items for Systematic Review and Meta-Analysis Protocols (PRISMA-P) 2015 statement. Syst Rev. 2015;4(1):1.

		Reporting Item	Page Number
**Title**			
Identification	#1a	Identify the report as a protocol of a systematic review	1
Update	#1b	If the protocol is for an update of a previous systematic review, identify as such	n/a

For peer review only

**Registration**			
	#2	If registered, provide the name of the registry (such as PROSPERO) and registration number	2
**Authors**			
Contact	#3a	Provide name, institutional affiliation, e-mail address of all protocol authors; provide physical mailing address of corresponding author	1
Contribution	#3b	Describe contributions of protocol authors and identify the guarantor of the review	13
**Amendments**			
	#4	If the protocol represents an amendment of a previously completed or published protocol, identify as such and list changes; otherwise, state plan for documenting important protocol amendments	n/a
**Support**			
Sources	#5a	Indicate sources of financial or other support for the review	13
Sponsor	#5b	Provide name for the review funder and / or sponsor	13
Role of sponsor or funder	#5c	Describe roles of funder(s), sponsor(s), and / or institution(s), if any, in developing the protocol	13
**Introduction**			

For peer review only

Rationale	#6	Describe the rationale for the review in the context of what is already known	3-6
Objectives	#7	Provide an explicit statement of the question(s) the review will address with reference to participants, interventions, comparators, and outcomes (PICO)	6
**Methods**			
Eligibility criteria	#8	Specify the study characteristics (such as PICO, study design, setting, time frame) and report characteristics (such as years considered, language, publication status) to be used as criteria for eligibility for the review	7
Information sources	#9	Describe all intended information sources (such as electronic databases, contact with study authors, trial registers or other grey literature sources) with planned dates of coverage	7
Search strategy	#10	Present draft of search strategy to be used for at least one electronic database, including planned limits, such that it could be repeated	7 (Supplementary file)
Study records - data management	#11a	Describe the mechanism(s) that will be used to manage records and data throughout the review	8
Study records - selection process	#11b	State the process that will be used for selecting studies (such as two independent reviewers) through	8

For peer review only

		each phase of the review (that is, screening, eligibility and inclusion in meta-analysis)	
Study records - data collection process	#11c	Describe planned method of extracting data from reports (such as piloting forms, done independently, in duplicate), any processes for obtaining and confirming data from investigators	8
Data items	#12	List and define all variables for which data will be sought (such as PICO items, funding sources), any pre-planned data assumptions and simplifications	8-9
Outcomes and prioritization	#13	List and define all outcomes for which data will be sought, including prioritization of main and additional outcomes, with rationale	9
Risk of bias in individual studies	#14	Describe anticipated methods for assessing risk of bias of individual studies, including whether this will be done at the outcome or study level, or both; state how this information will be used in data synthesis	9-10
Data synthesis	#15a	Describe criteria under which study data will be quantitatively synthesised	10
Data synthesis	#15b	If data are appropriate for quantitative synthesis, describe planned summary measures, methods of handling data and methods of combining data from studies, including any planned exploration of consistency (such as I2, Kendall's τ)	10

For peer review only

Data synthesis	#15c	Describe any proposed additional analyses (such as sensitivity or subgroup analyses, meta-regression)	10
Data synthesis	#15d	If quantitative synthesis is not appropriate, describe the type of summary planned	10
Meta-bias(es)	#16	Specify any planned assessment of meta-bias(es) (such as publication bias across studies, selective reporting within studies)	n/a
Confidence in cumulative evidence	#17	Describe how the strength of the body of evidence will be assessed (such as GRADE)	9

None The PRISMA-P checklist is distributed under the terms of the Creative Commons Attribution License CC-BY 4.0. This checklist can be completed online using https://www.goodreports.org/, a tool made by the EQUATOR Network in collaboration with Penelope.ai

For peer review only
